# Eye Muscle MRI in Myasthenia Gravis and Other Neuromuscular Disorders

**DOI:** 10.3233/JND-230023

**Published:** 2023-09-08

**Authors:** Kevin R. Keene, Irene C. Notting, Jan J.G.M. Verschuuren, N. Voermans, Ronald. O.B. de Keizer, Jan-Willem M. Beenakker, Martijn R. Tannemaat, Hermien E. Kan

**Affiliations:** a Department of Radiology, CJ Gorter MRI Center, Leiden University Medical Center, Leiden, The Netherlands; b Department of Neurology, Leiden University Medical Center, Leiden, The Netherlands; c Department of Ophthalmology, Leiden University Medical Center, Leiden, The Netherlands; d Department of Neurology, Radboud University Medical Center, Nijmegen, The Netherlands; e The Rotterdam Eye Hospital, Rotterdam, The Netherlands; f Department of Radiation Oncology, Leiden University Medical Center, Leiden, The Netherlands

**Keywords:** Myasthenia gravis, quantitative MRI, eye muscles, extra-ocular muscles, chronic progressive external ophthalmoplegia (CPEO), diplopia, ptosis, oculopharyngeal muscular dystrophy (OPMD) and Graves’ orbitopathy

## Abstract

**Introduction:**

MRI of extra-ocular muscles (EOM) in patients with myasthenia gravis (MG) could aid in diagnosis and provide insights in therapy-resistant ophthalmoplegia. We used quantitative MRI to study the EOM in MG, healthy and disease controls, including Graves’ ophthalmopathy (GO), oculopharyngeal muscular dystrophy (OPMD) and chronic progressive external ophthalmoplegia (CPEO).

**Methods:**

Twenty recently diagnosed MG (59±19yrs), nineteen chronic MG (51±16yrs), fourteen seronegative MG (57±9yrs) and sixteen healthy controls (54±13yrs) were included. Six CPEO (49±14yrs), OPMD (62±10yrs) and GO patients (44±12yrs) served as disease controls. We quantified muscle fat fraction (FF), T2_water_ and volume. Eye ductions and gaze deviations were assessed by synoptophore and Hess-charting.

**Results:**

Chronic, but not recent onset, MG patients showed volume increases (e.g. superior rectus and levator palpebrae [SR+LPS] 985±155 mm^3^ compared to 884±269 mm^3^ for healthy controls, *p* < 0.05). As expected, in CPEO volume was decreased (e.g. SR+LPS 602±193 mm^3^, *p* < 0.0001), and in GO volume was increased (e.g. SR+LPS 1419±457 mm^3^, *p* < 0.0001). FF was increased in chronic MG (e.g. medial rectus increased 0.017, *p* < 0.05). In CPEO and OPMD the FF was more severely increased. The severity of ophthalmoplegia did not correlate with EOM volume in MG, but did in CPEO and OPMD. No differences in T2_water_ were found.

**Interpretation:**

We observed small increases in EOM volume and FF in chronic MG compared to healthy controls. Surprisingly, we found no atrophy in MG, even in patients with long-term ophthalmoplegia. This implies that even long-term ophthalmoplegia in MG does not lead to secondary structural myopathic changes precluding functional recovery.

## INTRODUCTION

Myasthenia gravis (MG) is a muscle disease characterized by fluctuating and fatigable muscle weakness. MG is caused by auto-antibodies targeting proteins at the neuromuscular junction, including the acetylcholine receptor (AChR), muscle-specific tyrosine kinase (MuSK) or LRP4. [1, 2] Most patients experience ocular symptoms like diplopia and ptosis during the course of their disease [3, 44]. Both the diagnosis of ocular MG and the treatment of refractory ocular symptoms are major challenges in MG. In half of the ocular MG patients, no serum auto-antibodies against AChR, MuSK or LRP4 are found. Moreover, the sensitivity of repetitive nerve stimulation is low in ocular MG, and while single-fiber EMG has a higher sensitivity, it requires a specifically trained neurophysiologist to perform the measurement and is thus operator-dependent [1, 2, 5]. Ocular MG can therefore pose a significant diagnostic challenge [4, 6]. Furthermore, in some MG patients a therapeutic resistant ophthalmoparesis develops and little is known about its pathophysiology.

MRI of the extra-ocular muscles (EOM) can be used to directly assess structural changes in the EOM, and can therefore be of significance both for diagnostics in seronegative MG and for understanding the pathophysiology of therapy-resistant ophthalmoplegic MG. In skeletal muscles, quantitative MRI is widely used to study fat replacement, muscle size and T2 relaxation time changes [7]. Recently, we showed that quantitative MRI of individual EOM (lateral rectus[LR], medial rectus[MR], inferior rectus[IR] and superior rectus[SR]) and the levator palpebrae superioris muscle (LPS) in a small group of MG patients was feasible [8]. Diagnostically, MRI of the EOM is rarely performed in MG and little is known how structural EOM changes in MG relate to changes observed in other ocular diseases. For example, in Graves’ orbitopathy (GO), characteristic fusiform swelling of the EOM and adipogenesis in the EOM are observed on MRI, and volume correlates with disease stage [9]. Furthermore, in chronic progressive external ophthalmoplegia (CPEO) EOM are atrophic and EOM volume correlates with maximal range of eye movements [10].

MRI could also aid in understanding therapy resistant ophthalmoplegia in MG. Several case reports [11–14] and a study with a larger cohort [15] suggest that untreated MG and MuSK MG patients can have EOM atrophy. Atrophy is therefore a proposed mechanism of refractory ophthalmoplegia in MG [2, 16–18]. As such, studying to what extent atrophy occurs in the EOM, and what the influence of treatment is, could aid in pathophysiological understanding. Additionally, by correlating EOM function to structural changes with orthoptic tests, the influence of atrophy on function can directly be assessed. We have previously shown that orthoptic tests are sensitive to ’ MG related EOM weakness [19].

We hypothesize that in MG structural changes are present in the EOM. In the current study, we first aimed to identify structural EOM differences in a large group of MG patients using quantitative MRI parameters to aid in diagnostics. Secondly, we aimed to identify structural changes in EOM that could explain refractory ophthalmoplegia in ocular MG. To this end, untreated recently diagnosed, treated chronic and seronegative MG patients were included. Finally, we studied correlations between orthoptics and quantitative MRI parameters to determine whether structural changes were related to functional deficits.

## METHODS

### Participants

We included a convenience sample of MG, GO, CPEO and OPMD patients from the Neurology Department and the Ophthalmology Department of the LUMC, Radboud university and the Rotterdam Eye Hospital as described before. Given logistics and the COVID-19 pandemic including a consecutive sample of patients was not possible, however we asked patients as consecutively as possible. Age matched healthy controls were recruited using flyers and posters and by sending letters asking patients’ relatives. Detailed data from the orthoptic tests have been reported separately [19].

Three groups of autoimmune MG patients were included: recently diagnosed, chronic and seronegative. The diagnosis in chronic and recently diagnosed MG patients was based on the combination of typical muscle weakness and the presence of serum autoantibodies to AchR [1, 2, 4]. Seronegative myasthenia gravis (SNMG) was defined as clinically confirmed fluctuating muscle weakness in combination with abnormal decrement during repetitive nerve stimulation (RNS), increased jitter during single fiber EMG testing or a positive response to an acetylcholinesterase inhibitor, together with absence of AChR or muscle-specific kinase (MuSK) serum autoantibodies [4]. In recently diagnosed MG patients the diagnosis, as described above, was established less than a year ago and they were not treated with systemic immunosuppressants. Chronic MG patients received their diagnosis more than a year ago and no selection was made based on previous treatments. SNMG patients were selected regardless the disease duration or corticosteroid use in the past. We also included three other disease groups with EOM involvement: GO, CPEO and OPMD, and a group of healthy age and sex-matched controls. The diagnosis of GO was defined as the presence of TSH-receptor serum autoantibodies with presence of ocular symptoms [20]. The diagnosis of CPEO was confirmed by genetic testing or with skeletal muscle biopsy [21] and the diagnosis of OPMD was confirmed with genetic testing of the PABPN1 gene [22]. Participants with a history of strabismus were excluded. Patients with a simultaneous diagnosis of MG and GO were excluded. None of MG and none of GO patients had received orbital surgery or radiotherapy. Two of the CPEO and one of the OPMD patients had surgery involving the levator palpebrae to correct their ptosis.

For the MG patients a quantitative myasthenia gravis (QMG) score [23, 24] and a myasthenia gravis activities of daily living (MG-ADL) scale [25] were recorded.

### Standard protocol approvals, registrations, and patient consents

The Medical Ethics Review Committee Leiden Den Haag Delft approved the study and its use of human subjects. All patients provided informed, written consent prior to study participation.

### MR Examination

All subjects were scanned in supine position on 7T Philips Achieva MRI (Philips Healthcare, Best, The Netherlands) with the upper 16 elements of a 32-channel head-coil (Nova Medical). Using cued-blinking [26] the MRI acquisitions were halted periodically, and patients were visually instructed to blink. Scan-times were kept short, under 4 minutes, to reduce movement artefacts [27]. We performed a chemical shift based water fat separation gradient echo scan (from now on referred to as Dixon scan) to quantify muscle fat fraction and muscle volume [8] and Multi echo Spin-echo (MSE) to assess the T2 relaxation time of water (T2_water_), as an indicator of disease activity [28, 29].

### Volumes and fat fractions: Dixon scan

#### MR acquisition

A 3-point multi-acquisition 3D Dixon scan was acquired (resolution: 0.8×0.8×0.8 mm^3^, first time to echo (TE)/*Δ*TE/repetition time (TR)/flip-angle (FA)/scan time:2.4 ms/0.33 ms/8 ms/7°/3 : 50 min). DREAM B1 maps were acquired for the last 5 participants (3 slices, 1.2×1.2×3.0 mm^3^) [30].

#### Postprocessing

The real and imaginary echo data from the Dixon scan were used to reconstruct water and fat images using Iterative decomposition of water and fat (IDEAL) using an in-house developed Matlab script (Matlab 2016a, The Mathworks of Natick, Massachusetts, USA; https://git.lumc.nl/neuroscience/StandardizedDixonPipeline, without B0 field smoothing, T2* corrections or region growing algorithms [31].

#### Segmentation

The EOM were semi-automatically segmented on the water image in 3D using the seed growing algorithm of ITK-SNAP [32], which is possible given the distinct contrast between the EOM and the intra-orbital fat. After seed growing, possible intra-orbital arteries, veins and nerves were manually removed from the 3D volume of interest. The SR was segmented together with the levator palpebrae given the difficulty of segmenting them separately. This muscle complex is referred to as the *superior rectus plus levator palpebrae superioris* (SR+LPS). All segmentations were done by the same observer (KK).

#### Analysis

The water and fat images were used to calculate fat fraction maps. Corrections for T1 weighting were applied to the fat fractions per voxel for the Dixon scan using the Ernst angle equation using an effective flip angle and the repetition time of 8 ms [33]. As the B1 varies spatially, an EOM-specific effective flip angle of the 7 degree pulse was calculated based on the B1 measurements obtained in 5 participants. To this end, all eight recti EOM were segmented manually on the middle slice of the B1 map and EOM specific values were calculated by averaging the voxels in the regions of interest.

### T2_water_: Multi-echo spin-echo (MSE) Scans

#### MR acquisition

A MSE scan was acquired per orbit (resolution: 1.2×1.2×3.0 mm^3^, first TE/*Δ*TE/TR: 9 ms/9 ms/4000 ms, 24 echo’s, 3 slices per eye, scan time: 2 : 44 min, slice selection gradient strength: excitation 3.15 mT/m and refocusing 3.78 mT/m). This scan was planned with one orbit in the field of view perpendicular to the MR and LR muscles. In the T2_water_ analysis of skeletal muscle, the subcutaneous fat that is present in the field of view is conventionally used to calibrate the T2 of the fat compartment (T2_fat_). However, no subcutaneous fat is present in the field of view in the acquired orbital scans. Therefore, for calibration of the T2_fat_ an additional MSE neck scan was acquired and T2_fat_ was calibrated on subcutaneous fat of the neck. In the last three echoes of both acquisitions, the RF-pulses were disabled.

#### Postprocessing

The MSE scans were analyzed using a two-component Extended Phase Graphs (EPG) model, consisting of a water and a fat component, as described before with corrections for the flip-angle slice profile [28]. The T2 of the fat compartment was calibrated on subcutaneous fat of the neck. The dictionary used for fitting contained T2_water_ values from 10 ms to 60 ms, T2_fat_ values from 120 ms to 200 ms and B1 values from 50% to 100%. Water, fat, fat fraction, T2_water_ and residual maps were exported. An in-house developed Matlab script was used to perform this dictionary fitting(https://git.lumc.nl/neuroscience/multicomponent_t2_epg).

#### Segmentation

The EOM were segmented by drawing regions of interest polygons per slice using the water map as an anatomical reference by one observer (KK).

#### Analysis

The EOM specific T2_water_ values were calculated by averaging the voxels in the regions of interest for all slices after erosion of one voxel.

### Orthoptic measurements

#### Measurements

Duction angles were defined as the range of motion of the eye in degrees in all eight cardinal gaze directions (horizontal, vertical and diagonal). In this study, unilateral duction angles were determined using the synoptophore (Clement Clarke International, 2002, Edinburgh way, Harlow, Essex). Elevation/depression was measured up to+/- 30° and abduction/adduction was measured up to+/- 40°. Gaze deviations between eyes were measured using standard Hess-chart examination [34]. Both measurements were performed by a single not masked observer (KRK). To recapitulate, the synoptophore measures limited ductions for one eye at the time, while the Hess-chart examination is based on the testing of deviations between both eyes in different gaze directions. The orthoptic studies were performed contiguous with the MRI on the same day. Given logistic problems patients were asked but not obliged to refrain from pyridostigmine use. The patients who used pyridostigmine generally took it in the morning at home before travelling to the hospital, they were then scanned at noon and the orthoptic evaluation was contiguous at one o’clock. Therefore in most patients the pyridostigmine effect was worn out before testing. However we did not structurally record the times.

#### Analysis

The EOM of MG patients were divided in ‘affected’ and ‘not affected’ for both orthoptic tests: A group of EOM with and without any duction limitations and a group of EOM with or without deviations above 5 degrees on the Hess-chart. The cut-off point of 5 degrees is commonly used in orthoptic clinical practice. The duction limitation or Hess-chart deviation in the primary direction of action of specific EOM was used (e.g. abduction for the LR and adduction for the MR). Additionally, we defined the severity of ophthalmoplegia per patient as the sum of duction limitations in all directions for both angles. This sum-score was used to study correlations between EOM volume and the severity of ophthalmoplegia.

### Statistical analysis

EOM specific values were compared between disease groups and myasthenia groups using repeated measures ANOVA defining laterality (left and right eye) as a repeated measure to assess differences between all groups that could be used to aid in diagnostics. *Post-hoc* testing was performed using Dunnett’s multiple comparisons test. For the second aim, correlation within and between quantitative MRI measures and the continuous variables age and duction limitations were analyzed using Pearson correlations. For age and functional correlation the EOM measures were combined and volume was normalized to the mean of the healthy control per individual EOM, to correct for anatomical differences in size between different EOM (e.g., the SR+LPS is on average bigger than the LR). For volume, fat fraction and T2_water_ the amount of patients with zero, one or more than one muscle higher or lower than 2 standard deviations (SD) above and below the mean of healthy controls were calculated. Statistical analysis was performed with SPSS version 23 (IBM Corp, Armonk, NY) and *p* values below 0.05 were considered significant.

### Data availability

The authors confirm that the data supporting the findings of this study are available within the article and its supplementary material (supplementary Table 1). This data is made available strictly for academic usage and not for commercial purposes.

## RESULTS

**Fig. 1:**
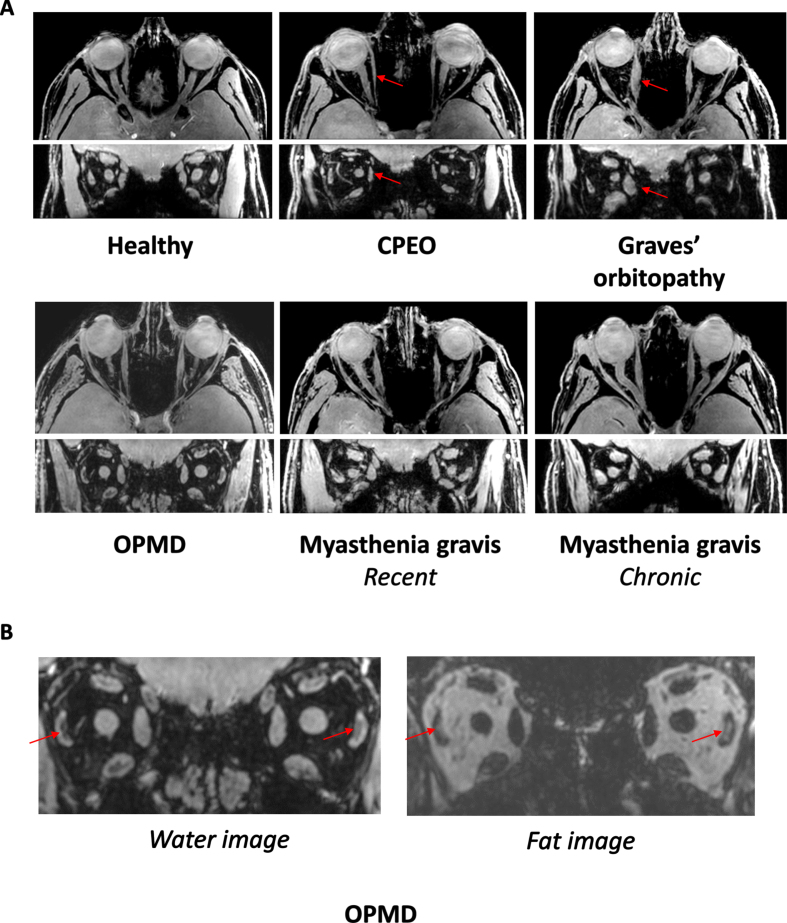
A. MRI scans from the orbit showing the extra-ocular muscles in a healthy control, a chronic progressive external orbitopathy (CPEO) patient, a Graves’ orbitopathy patient, an oculopharyngeal muscular dystrophy (OPMD) patient, a recent myasthenia gravis patient and a chronic myasthenia gravis patient. The axial and coronal water images are shown from a chemical shift based water-fat separation gradient echo scan, a technique to separately image and quantify water and fat. The red arrows indicate the atrophic medial rectus muscle in the CPEO patient and the swollen medial rectus muscle in the Graves’ orbitopathy patient. B. Example of fat replacement of the lateral rectus muscles in an OPMD patient as depicted by the red arrows. On the left the coronal water image and on the right the coronal fat image are shown.

Examples of axial and coronal orbital water images from Dixon scans are shown in Fig. 1A for a healthy control and in Fig. 1C for CPEO, GO, a recent MG and a chronic MG patient. An example of fat replacement in an OPMD patient is shown in Fig. 1B.

### Participant characteristics

We included 16 healthy controls, 20 recently diagnosed MG patients, 19 chronic MG patients, 14 SNMG patients, 6 CPEO patients, 6 OPMD patients and 6 GO patients. MRI data could not be acquired in four participants due to claustrophobic symptoms before scanning and in two participants due to a faulty amplifier resulting in poor scan quality. These six patients (three seronegative MG, one recent MG, one chronic MG and one GO patient) were excluded from the analyses. In four MG patients no neck reference MSE scan was obtained due to time constraints, therefore these patients were excluded from the T2_water_ analysis. Demographic and clinical baseline characteristics of all participants are shown in Table 1. No significant differences were found between the baseline characteristics sex and age between all groups.

**Table 1:** Baseline characteristics and sum-scores of 87 participants included in this study: 16 healthy controls, 20 recently diagnosed myasthenia gravis (MG) patients, 19 chronic MG patients, 14 seronegative MG (SNMG) patients, 6 chronic progressive external ophthalmoplegia (CPEO) patients, 6 oculopharyngeal muscular dystrophy (OPMD) patients and 6 Graves’ orbitopathy (GO) patients. Data are presented as number of patients (%) for categorical variables and as mean±SD for continuous variables. Synoptophore abnormalities is defined as having limited duction angles in any direction in one or both eyes. Hess-chart abnormalities is defined as having gaze deviations between the eyes above 5 degrees in any direction

	**MG** *Recently diagnosed* *n* = 20	**MG** *Chronic* *n* = 19	**MG** *Seronegative* *n* = 14	**CPEO** *n* = 6	**OPMD** *n* = 6	**GO** *n* = 6	**Healthy controls** *n* = 16	***p-value***
**Age (yrs)**	59±19	51±16	57±9	49±14	62±10	44±12	54±13	*0.243*
**Sex**								*0.754*
*Female*	7 (35%)	9 (47%)	7 (50%)	3 (50%)	4 (67%)	4 (67%)	9 (56%)	
*Male*	13 (65%)	10 (53%)	7 (50%)	3 (50%)	2 (33%)	2 (33%)	7 (44%)	
**Phenotype**								*0.105*
*Ocular*	12 (60%)	6 (32%)	9 (64%)	-	-	-	-	
*Generalized*	8 (40%)	13 (68%)	5 (36%)	-	-	-	-	
**Disease duration (months)**	4.0±2.2	75.6±87.9	25.6±60.5	-	-	22.8±35.9*	-	< *0.0001*
**MG-ADL**	5.8±3.3	5.5±4.2	5.0±2.7	-	-	-	-	*0.791*
**MG-ADL (ocular items)**	2.8±1.1	1.7±0.8	2.9±1.2					*0.165*
**QMG**	9.2±6.0	9.8±7.7	8.3±4.6	-	-	-	-	*0.812*
**QMG (ocular items)**	2.2±0.7	1.2±0.7	2.5±1.0					*0.365*
**Pyridostigmine on study day**	25%	15%	14%	-	-	-	-	0.680
**Hess-chart abnormalities**	15	15	11	6	5	5	0	
**Synoptophore abnormalities**	9	10	7	6	5	1	0	
**Ptosis at visit**	10	5	5	5	5	0	0	

^*^Two Graves’ orbitopathy patients were diagnosed longer than>one year ago and four patients<one year ago.

### Muscle volumes differ between disease groups

**Fig. 2:**
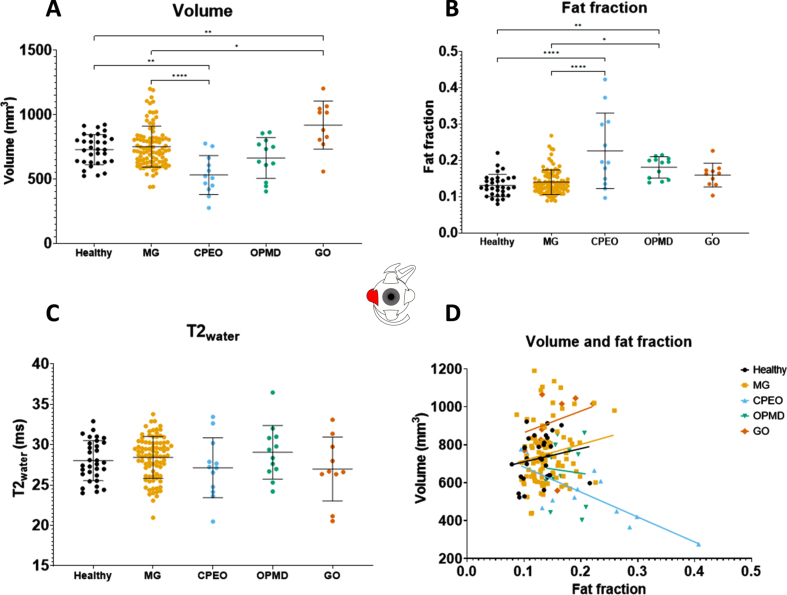
Volume values, fat fraction values, T2_water_ values and the correlation between fat fraction and volume of the **lateral rectus** as measured with quantitative MRI for the different groups: healthy controls, myasthenia gravis (MG), chronic progressive external ophthalmoplegia (CPEO), oculopharyngeal muscular dystrophy (OPMD) and Graves’ orbitopathy. (GO). ^*^is *p*≤ 0.05; **is *p*≤0.01; ^***^is *p*≤  0.001; ^****^is *p*≤  0.0001.

**Fig. 3:**
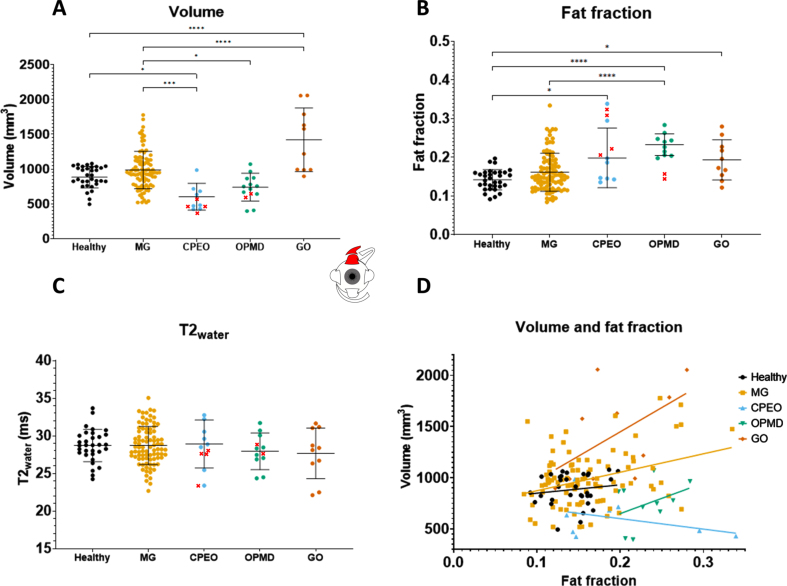
Volume values, fat fraction values, T2_water_ values and the correlation between fat fraction and volume of the **superior rectus plus the levator palpebrae** as measured with quantitative MRI for the different groups: healthy controls, myasthenia gravis (MG), chronic progressive external ophthalmoplegia (CPEO), oculopharyngeal muscular dystrophy (OPMD) and Graves’ orbitopathy (GO). The values marked with a red cross in OPMD and CPEO are from patients who had eyelid corrective surgery involving the levator palpebrae and were excluded from the mean. ^*^is *p*≤0.05; ^**^is *p*≤0.01; ^***^is *p*≤0.001; ^****^is *p*≤  0.0001.

**Fig. 4:**
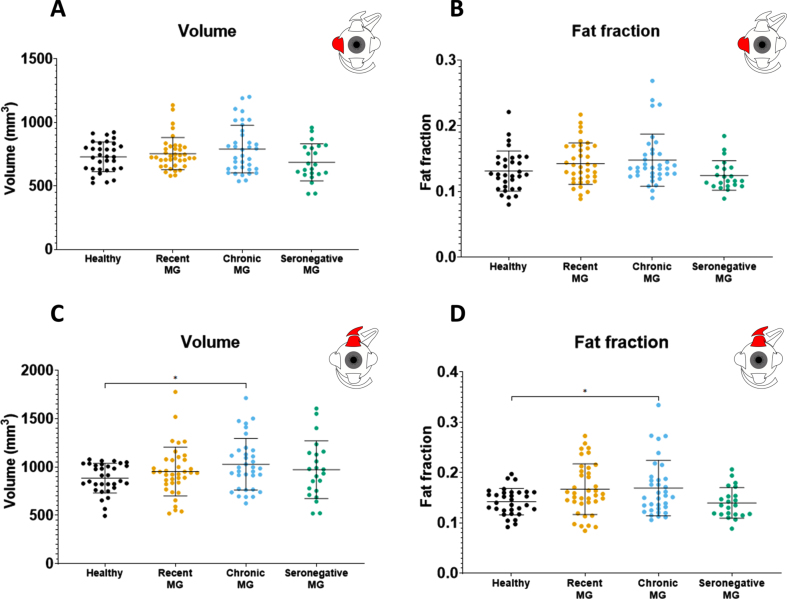
Volume values and fat fraction values for **the lateral rectus and the superior rectus plus levator palpebrae** as measured with quantitative MRI for the different myasthenia gravis groups as compared to healthy controls: recently diagnosed myasthenia gravis, chronic myasthenia gravis and seronegative myasthenia gravis. ^*^is *p*≤0.05; ^**^is *p*≤0.01; ^***^is *p*≤0.001; ^****^is *p*≤0.0001.

Volume differences were observed between disease groups (Table 2). No volume differences were observed between the combined MG patient cohort and healthy controls in all recti EOM. For example, the volume for the LR was 728.1± 116.8 mm^3^ in healthy controls and 751.2± 159.6 mm^3^ in MG (Fig. 2A) and the volume for the SR+LPS was 883.8± 268.5 mm^3^ in healthy controls and 984.7± 153.7 mm^3^ in MG (Fig. 3A). Differences were observed between MG patients and CPEO (*p* < 0.0001), OPMD (*p* < 0.05) and GO (*p* < 0.0001) patients in the SR+LPS (Fig. 3A). The LR volume was different between MG and CPEO (*p* < 0.0001) and GO (*p* < 0.05, Fig. 2A). The MR volume differed between MG and CPEO (< 0.01) and the IR volume between MG and GO (*p* < 0.0001). When looking at the subgroups of MG patients, volume was increased in chronic MG patients as compared to healthy controls in the SR+LPS (*p* < 0.05 Fig. 4) and the IR (*p* < 0.05). Volume was not different in recently diagnosed and seronegative MG patients for all EOM.

**Table 2:** The quantitative MRI parameters (volume, fat fraction and T2_water_-) for all individual extra-ocular muscles for the MG groups recently diagnosed, chronic and seronegative, for the disease controls chronic progressive external ophthalmoplegia (CPEO), oculopharyngeal muscular dystrophy (OPMD) and Graves’ orbitopathy (GO), and the healthy controls. For volume, fat fraction and T2_water_ the amount of patients with zero, one or more than one muscle higher or lower than 2 standard deviations (SD) above and below the mean of healthy controls are mentioned. Per quantitative MRI parameter an ANOVA was performed and for the group comparison a *post-hoc* testing was performed using Dunnett’s multiple comparisons test to correct for multiple testing

	**MG** *Recently diagnosed* *n* = 19	**MG** *Chronic* *n* = 18	**MG** *Seronegative* *n* = 11	**CPEO** *n* = 6	**OPMD** *n* = 6	**GO** *n* = 5	**Healthy controls** *n* = 16	*p-value with MG groups combined*
**Volume**								
*Lateral rectus* (mm^3^)	753.5±126.7	753.5±187.6	685.0±145.7	**531.0**±116.8^*^	662.6±158.5	**918.6**±186.2^*^	728.1±116.8	< *0.0001*
*Medial rectus* (mm^3^)	664.8±117.6	691.5±154.0	569.5±101.5	**497.1**±121.3^*^	618.0±103.8	**775.6**±188.5^*^	629.8±142.2	*0.0002*
*Inferior rectus* (mm^3^)	517.3±121.3	**565.4**±156.8^*^	408.9±124.4	432.4±134.5	546.3±164.9	**857.1**±424.0^*^	480.4±134.5	< *0.0001*
*Superior rectus plus levator palpebrae* (mm^3^)	953.1±252.8	**1028.0**±267.7^*^	972.4±298.5	**602.0**±192.9^*^†	739.8±199.8†	**1419.0**±457.1^*^	883.8±153.7	< *0.0001*
*0 muscles out of 2 SD range (n [% ])*	13 (68%)	8 (44%)^*^	5 (46%)	2 (33%)^*^	3 (50%)	0 (0%)^*^	11 (69%)	< *0.01*
*1 muscles out of 2 SD range (n [% ])*	2 (11%)	4 (22%)^*^	4 (36%)	0 (0%)^*^	2 (33%)	0 (0%)^*^	5 (31%)	
*2* + *muscles out of 2 SD range (n [% ])*	4 (21%)	6 (33%)^*^	2 (18%)	4 (67%)^*^	1 (17%)	5 (100%)^*^	0 (0%)	
**Fat fraction**								
*Lateral rectus*	0.142±0.032	0.147±0.040	0.114±0.022	**0.226**±0.104^*^	**0.181**±0.029^*^	0.160±0.033	0.131±0.031	< *0.0001*
*Medial rectus*	0.135±0.028	**0.140**±0.030*	0.137±0.026	**0.202**±0.061^*^	**0.163**±0.018^*^	0.142±0.028	0.123±0.019	*0.0075*
*Inferior rectus*	0.174±0.052	0.187±0.046	0.151±0.042	**0.223**±0.057^*^	0.194±0.035	0.199±0.077	0.169±0.045	< *0.0001*
*Superior rectus plus levator palpebrae*	0.167±0.050	**0.170**±0.055^*^	0.140±0.030	**0.199**±0.077^*^†	**0.233**±0.028^*^†	**0.194**±0.052^*^	0.142±0.026	< *0.0001*
*0 muscles out of 2 SD range (n [% ])*	10 (53%)	9 (50%)^*^	7 (64%)	2 (33%)^*^	1 (17%)^*^	2 (40%)	13 (81%)	< *0.01*
*1 muscles out of 2 SD range (n [% ])*	5 (26%)	4 (22%)^*^	4 (36%)	0 (0%)^*^	0 (0%)^*^	2 (40%)	3 (19%)	
*2* + *muscles out of 2 SD range (n [% ])*	3 (16%)	5 (28%)^*^	0 (0%)	4 (67%)^*^	5 (83%)^*^	1 (20%)	0 (0%)	
**T2**_**water**_								
*Lateral rectus (ms)*	28.4±2.2	28.7±2.5	28.0±3.1	27.1±3.7	29.1±3.3	27.0±4.0	28.0±2.5	*n.s.*
*Medial rectus (ms)*	26.3±2.9	28.3±3.3	28.3±3.3	28.5±3.2	26.4±1.8	26.6±4.9	27.0±2.6	*n.s.*
*Inferior rectus (ms)*	24.5±3.6	25.8±3.4	26.8±3.9	24.7±3.0	**29.3**±2.7*	25.1±6.2	24.3±4.0	*0.0053*
*Superior rectus plus levator palpebrae (ms)*	27.7±2.1	29.1±2.5	29.5±2.7	28.9±3.2	28.0±2.4	27.7±3.4	28.7±2.1	*n.s.*
*0 muscles out of 2 SD range (n [% ])*	13 (68%)	12 (67%)	5 (46%)	1 (16.5%)	4 (67%)	1 (20%)	11 (69%)	n.s.
*1 muscles out of 2 SD range (n [% ])*	6 (32%)	4 (22%)	4 (36%)	4 (67%)	2 (33%)	2 (40%)	5 (31%)	
*2* + *muscles out of 2 SD range (n [% ])*	0 (0%)	2 (11%)	2 (18%)	1 (16.5%)	0 (0%)	2 (40%)	0 (0%)	

^*^The bold values are significantly different from healthy controls after *post-hoc* testing. †Two of the CPEO and one of the OPMD patients had surgery involving the levator palpebrae to correct for their ptosis and excluded from the mean.

### Fat fractions are higher in CPEO, OPMD and chronic MG

Fat fraction differences were observed between disease groups (Table 2). No fat fraction differences were observed between the combined MG patient cohort and healthy controls. For example, the fat fraction of the LR was 0.131± 0.031 for healthy controls and 0.140± 0.034 for all MG groups combined (Fig. 2B) and for the SR+LPS the fat fraction was 0.142± 0.026 in healthy controls and 0.162± 0.049 in MG (Fig. 3B). Differences were observed between MG patients and CPEO for all EOM (Fig. 2B). Between MG and OPMD the fat fraction differed for the IR (*p* < 0.05) and the SR+LPS (*p* < 0.05). When looking at subgroups of MG patients, differences in fat fraction were observed between chronic MG patients and healthy controls in the MR (0.017 difference, *p* < 0.05) and SR+LPS (0.028 difference, *p* < 0.05), but not for recently diagnosed and seronegative MG patients.

### Volume correlates with fat fraction in MG, CPEO and graves’ orbitopathy

In MG patients, volume and fat fraction correlated moderately in MR, IR and SR+LPS muscles (e.g. for SR+LPS *r* = 0.27, *p* <0.001, Fig. 3D): bigger EOMs had higher fat fractions, with an average difference of 5% between a SR+LPS of 500 mm^3^ and 1500 mm^3^. In CPEO, a decrease in muscle volume was accompanied by an increase in fat fraction in the LR (*r* =–0.79, *p* < 0.01, Fig. 2D) and the SR+LPS (*r* =–0.61, *p* < 0.05, Fig. 3D). In OPMD, volume and fat fraction did not correlate and only the volume of the IR strongly correlated positively with fat fraction in GO (*r* = 0.81, *p* <0.01).

### Volume correlates with age in MG

Combining all EOM, we observed a negative correlation between age and normalized volume in MG patients (*r* =–0.25, *p* <0.01) but not in healthy controls (*r* =–0.02, n.s.). This difference might however be explained by the fact that a portion of the MG patient received immunosuppressant treatment including corticosteroids. Additionally a positive correlation between age and fat fraction was observed in healthy controls (*r* =0.17, *p* <0.05) and in MG patients (*r* =0.28, *p* <0.0001).

### All patient groups show limited ductions and hess-chart abnormalities

Limited ductions were observed in CPEO (6 out of 6) and OPMD (5 out of 6) patients (Table 1). In 26 of 53 MG patients, limited duction were found, which was comparable in chronic MG (10 out of 19), recent MG (9 out of 20) and seronegative MG (7 out of 14). Hess-chart abnormalities were observed in almost all patient control groups (6/6 in CPEO, 5/6 in OPMD and 5/6 in GO). In MG 41 out of 53 patients had Hess-chart abnormalities, meaning that 80% of included MG patients had clinical or subclinical diplopia.

### qMRI parameters do not correlate with orthoptic measures in MG

**Fig. 5:**
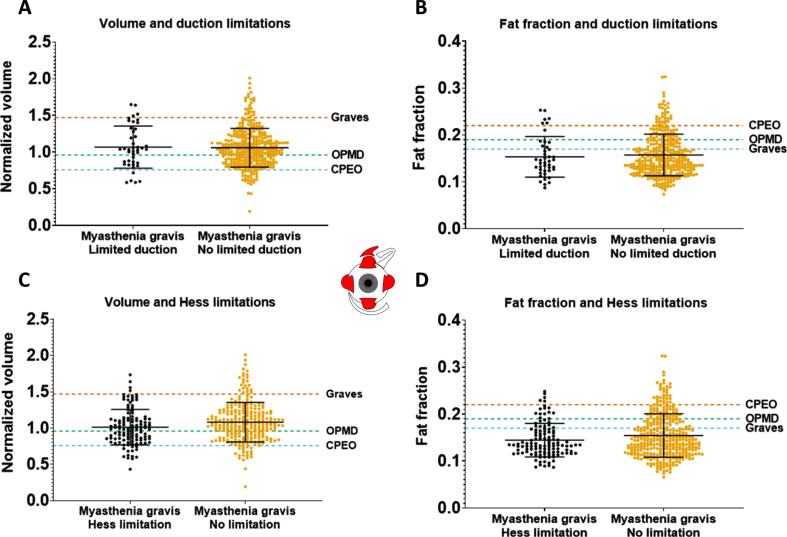
Volume and fat fraction for individual extra-ocular muscles in muscles with or without limited function, as measured with synoptophore (duction angles) and the outer field of the Hess-chart in myasthenia gravis patients. The averages of CPEO, OPMD and Graves’ orbitopathy patients are shown as dashed lines as a reference for higher/lower volume and fat fraction values. No significant differences were observed.

In all MG patients, volumes and fat fractions of individual EOM were compared in patients with or without limited duction angles. Additionally, a similar comparison was made between patients with and without deviations on the outer field of the Hess-chart. EOM responsible for duction limitations did not have a significantly different normalized volume or fat fraction than EOM not responsible for duction limitations as shown in Fig. 5A and 5B. In addition, the normalized volume and fat fraction of the EOM that showed a deviation on the Hess-chart were comparable to the normalized volume and fat fraction of EOM that did not (Fig. 5C and 5D).

The severity of ophthalmoplegia on a patient level was defined as the sum-score of limited ductions in all directions. This sum-score did not correlate with volume in MG for all EOM. In CPEO, a significant negative correlation between volume and this sum-score was observed in the LR (*r* =–0.79, *p* < 0.01), MR (*r* =–0.75, *p* < 0.01) and SR (*r* =–0.63, *p* < 0.05). Additionally, in OPMD a significant negative correlation between volume and this sum-score was present in the MR (*r* =–0.71, *p* < 0.05), LR (*r* =–0.76, *p* < 0.01) and SR (*r* =–0.58, *p* < 0.05). In GO a negative relationship was only significant in the LR (*r* =–0.65, *p* < 0.05).

We compared the sum-score of duction limitations between different groups based on the number of muscles with volume and fat fraction below/over two standard deviations from the healthy controls (zero, one or more than one). For volume, MG patients had an average sum-score of 7.4 degrees when zero muscles were out of the 2SD range, 12.4 degrees when one muscle was out of range and 38 degrees when more than two muscles were out of range (*p* < 0.05).

### T2_water_ is comparable between mg and healthy controls

No significant differences in T2_water_ for all individual EOM were found between MG patients and healthy controls and between MG groups (Fig. 2C, Fig. 3C and Table 2). Only for the IR, significant differences were found between OPMD (29.3± 2.7 ms) and CPEO (24.7± 3.0 ms, *p* < 0.05), MG (25.6± 3.7 ms, *p* < 0.05) and healthy controls (24.3± 4.0 ms, *p* < 0.01). Relatively large standard deviations for T2_water_ were observed for all groups including healthy controls (2.5 ms, 2.6 ms, 4.0 ms and 2.1 ms, respectively for LR, MR, IR and SR+LPS) for all EOM.

## DISCUSSION

In this work we systematically evaluated quantitative MRI and orthoptic tests to measure both structure and function of the EOM in a large and well-defined cohort of MG patients, neuromuscular controls and healthy controls. We found no atrophy and limited fat replacement in MG, even in patients with residual ophthalmoplegia. Instead, volume and fat fraction of EOM were slightly increased in chronic MG patients, which raises pathophysiological questions. The observed differences in EOM volume and fat fraction in CPEO, OPMD and GO validate MRI as a sensitive technique to study EOM. MRI of the EOM is generally not helpful to differentiate recent onset, chronic or seronegative MG from healthy controls in individual patients. In individual cases MRI of the EOM could be supportive in differential diagnostics of MG as compared to CPEO, OPMD and Graves. Our observations suggest that in most MG patients with refractory EOM weakness, no structural anatomical changes are present in the EOM precluding functional recovery after optimal treatment.

Our results show that MRI is not of immediate benefit for distinction between MG and healthy subjects. Muscle volume and FF were not different between healthy controls and seronegative MG patients, the subgroup which often poses diagnostic challenges. However, in individual cases orbital MRI can be useful to distinguish MG from common MG mimics like GO [35], OPMD and CPEO. In the latter three disorders the pattern of EOM involvement may be a diagnostic imaging marker, as earlier proposed by Ferreira et al. [36] In CPEO, the SR+LPS was most fat replaced and decreased in volume followed by the MR and LR, with relative sparing of the IR, which is in line with literature, pathophysiology and the clinical involvement pattern [10, 12]. In OPMD, the SR+LPS was also most fat replaced as expected [22, 37], followed by the LR and IR with relative sparing of the MR. In GO, EOM volume increased up to 190% without significant fat fraction increases, in line with previously described MRI and ultrasound measures described [9, 38]. Apparently, edematous swelling of EOM and adipogenesis result in increase in the amount of water and fat [39]. The involvement pattern was comparable to literature with predominant involvement of IR, SR+LPS and MR muscles [9, 36].

The unexpected absence of atrophy in MG is an important finding, as it excludes major structural changes in the muscles as a relevant factor in the pathophysiology of refractory ocular MG. In MG, EOM volumes and fat fractions were similar to healthy controls for most patients. In diseases with denervation like chronic fibrosis of the EOM (CFEOM) [40, 41] atrophy is observed. Similarly, we expected a decrease in muscle volume in chronic MG, especially in treatment refractory patients, due to synaptic denervation caused by the antibody-mediated neuromuscular junction damage and as observed in several case reports [11–14]. Given the absence of atrophy, it appears that long lasting functional denervation does not lead to EOM atrophy, in contrast to skeletal muscles. Possibly the unique innervation pattern with mono- and multisynaptic muscle fibers in the EOM can partly explain this [42–44] One case report also found no histological abnormalities in the EOM of a severely affected MG patient [45]. Several other case reports [11–14] on refractory AChR MG reported atrophy and fat replacement. In another study including severely refractory and untreated ocular MG patients [15] atrophy and fat replacement was reported, with a decrease in mean EOM thickness of 0.3mm and fat replacement as scored qualitatively. However, in that study, cross-sectional area was measured instead of volume, which can be affected by gazing direction and measurement location. The difference between this untreated refractory cohort and the chronic MG patients in the present study might also be explained by treatment effect, as all chronic MG patients in our study were treated with immunosuppressant medication drugs. This suggests a beneficial effect of immune suppressant treatment to prevent EOM atrophy. Moreover, the absence of gross structural muscle changes suggests that receptor blockage or damage at the neuromuscular junction are the main causes of ophthalmoplegia. This might imply that new treatments directed at the neuromuscular junction might still be able to reverse this refractory weakness.

In chronic MG patients the average EOM volume was significantly increased by approximately 20%. One possible explanation is a hypertrophic muscular response, as observed in other chronic muscular diseases like Duchenne muscular dystrophy [46] and in Charcot-Marie-Tooth disease type 1A [47]. Complement-mediated inflammation at the neuromuscular junction could possibly cause edema of muscle fibers [48]. Additionally, the copresence of fat in enlarged muscles in chronic MG suggests the presence of muscle damage, which may be secondary to muscle inflammation. We note that the chronic MG patients were relatively affected with a high MG-ADL and QMG (Table 1), this might be due to selection bias from being a tertiary referral center as an academic center and we included patients that came on the outpatient clinic with symptoms.

Although there were distinct differences in orthoptic measurements between patients and groups, in chronic MG patients with residual ophthalmoparesis the EOM were not atrophic. Additionally, there was no correlation between volume and fat fraction, and the orthoptic functional measures of individual EOM. On the other hand, in CPEO and OPMD a decrease in volume did correlate with orthoptic measures of EOM weakness. In these myopathic diseases, smaller EOM showed more limitations in eye movement. This has previously been reported for CPEO, where muscle size and T2 relaxometry correlated with range of eye movement [10]. The EOM of CPEO patients also show more volume decrease and FF increase than OPMD, which also fits with the clinical pattern with more presence of ophthalmoparesis in CPEO and more ptosis in OPMD. Altogether, this also shows that our methods were sensitive enough to detect correlations between EOM function and quantitative MRI. We did not structurally document whether the patients had ocular symptoms in the past. Therefore some MG patients without ocular symptoms at the time of the study could have had ocular involvement in the past, which might have resulted in structural changes in the EOM at the time of the MRI.

We observed a large, ∼3 ms, variation in T2_water_ of the EOM in healthy controls, which is larger than the average 1 ms previously observed in skeletal muscles [28, 29]. This might have masked the small expected increases in T2_water_ of 3 to 5 ms associated with minor inflammation [29]. Validation of the methodology on the semispinalis capitis, a major cervical muscle, in the same healthy volunteers showed lower variation in T2_water_ of 0.89 ms (supplementary material “Assessment of large variation in T2_water_”). We therefore performed a set of additional analyses on the potential origin of high variation observed in the EOM. These analyses included an assessment of the accuracy of EPG-based B1 determination and the influence of surrounding orbital fat that was superimposed on the MR-signal of the EOM. Similar to a recent 3T study on intra-ocular tumors [49], we observed a relatively low B1 in the EOM. Additionally, the T2 of the orbital fat appeared to be lower than the subcutaneous fat conventionally used as reference. However, correcting for these EOM-specific effects did not resolve the high variation in T2_water_. As a result, the origin of the high T2_water_ variation in the EOM remains unknown. It could, for example, have been a limitation of the two-component EPG model which does not incorporate a, currently unknown, EOM specific contribution to the MR-signal. Alternatively, the T2_water_ of the EOM could indeed have had a relatively high variation between subjects, making it a less useful metric to assess subtle changes due to inflammation.

For a better understanding of the pathophysiology of EOM changes in MG, a next step would be to include a group of patients with MuSK MG and congenital myasthenia syndrome. In these subgroups, we hypothesize that the EOM are atrophic due to the long lasting deficits in neuromuscular junction transmission, as has been shown for skeletal muscles in certain subtypes of congenital myasthenic syndrome using MRI [50]. Knowing whether EOM atrophy is present in these subgroups might aid in understanding how MG pathology behaves differently in EOM as compared to skeletal muscles. Additionally, microscopic evaluation of damage at the neuromuscular junctions in the EOM of MG patients may shed light on the pathophysiology of refractory ophthalmoparesis in chronic MG patients [18].

## FUNDING AND/OR CONFLICTS OF INTERESTS/COMPETING INTERESTS

K.R. Keene reports involvement in myasthenia gravis research sponsored by Argenx, Alexion Pharmaceuticals, and the CHDR, with all reimbursements received by the Leiden University Medical Center.

JJGMV has been involved MG research sponsored by the Princes Beatrix Fonds, Health Holland and consultancies for Argen-X, Alexion, and NMD Pharma. Reimbursements were received by the LUMC. He is coinventor on patent applications based on MuSK-related research. The Leiden University Medical Center receives royalties for MuSK antibody assays. He is a member of the European Reference Network for Rare Neuromuscular Diseases [ERN EURO-NMD]

M.R. Tannemaat reports trial support from ArgenX and Alexion, consultancies for ArgenX and UCB Pharma and research funding from NMD Pharma, with all reimbursements received by the Leiden University Medical Center. He is co-inventor on a patent describing a deep learning application to diagnose and monitor myasthenia gravis based on facial video data. J.W.M. Beenakker reports no disclosures.

I.C. Notting reports no disclosures.

H.E. Kan reports Trial Support from ImagingDMD, Reimbursements were received by the Leiden University Medical Center

N. Voermans reports no disclosures.

R.O.B. de Keizer reports no disclosures.

The C.J. Gorter MRI Center receives research support from Philips Healthcare.

## Supplementary Material

Supplementary Table 1

Supplementary Material
