# Supplementary material for: Eye Muscle MRI in Myasthenia Gravis and Other Neuromuscular Disorders
Source: J Neuromuscul Dis. 2023 Sep 8;10(5):869–83. doi: 10.3233/JND-230023 (PMC10578256; doi:10.3233/JND-230023)
Supplement: Supplementary Material [file jnd-10-jnd230023-s002.docx]

**Supplementary material – Appendix B: Assessment of the origin of the large variation in T2_water_ of the extra ocular muscles**

As we observed a relatively high variation in T2_water_ of the extra ocular muscles (EOM) (e.g. a standard deviation of 2.5 ms for the lateral muscle of healthy controls), we performed a set of additional analyses to assess its potential origin and evaluated different approaches to reduce it:

1. Validate the full method on a skeletal muscle
2. Effect of the surrounding orbital fat
3. Accuracy of EPG-based B1 determination
4. Effect of noise
5. EOM specific EPG analysis

**1) Validate full method on a skeletal muscle**

To assess if any of the specific details of the used methodology was responsible for the high variation in T2_water_, both in terms of scanning protocol and analysis, we analyzed data of the semispinalis capitis, a major muscles of the cervical spine, of the healthy volunteers (figure 1 left), which was acquired in the same scanning session, with the same protocol. We used the same EPG pipeline as used for the EOM, with the same T2 of the fat component obtained from the subcutaneous fat from the neck.


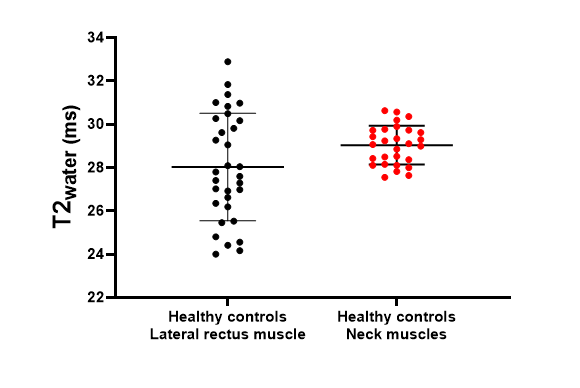

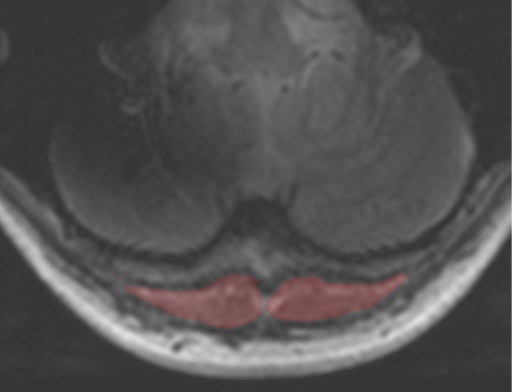
**Result:** For the “neck” muscle, we saw a significant lower variation in T2_water_ between subjects of 0.89 ms (figure 1 right), which is in line with other reports of the EPG-based T2_water_ mapping of skeletal muscles (e.g. 1.4 ms in the leg muscles^1^).

*Figure 1. Left: The segmentation of the semispinalis capitis muscle, a major muscle of the cervical spine. Right: The T2_water_ the later rectus EOM and the semispinalis capitis muscle of 16 healthy controls, using the original EPG analysis.*

**Conclusion**: The used methodology provided a reliable estimation of the T2_water_ in skeletal muscles and the observed higher variation is, at least partly, anatomy related.

**2) Effect of the surrounding orbital fat**

Due to the conical shape of the EOM and the chemical shift displacement of the fat signal, both in the read-out direction as in the slice direction, part of the fat signal was superimposed on the water signal of the EOM. At 7 Tesla this effect was pronounced compared to 3 Tesla, resulting in a 6 mm out of plane displacement in the scans used in this study. Although the used EPG-method as designed to separate these components, it relies on the assumption that the fat signal acquired in calibration step, in this case from the subcutaneous fat at the neck, has the same magnetic properties, especially T2_fat_, as the orbital fat.

To assess the validity of this assumption, we performed an EPG analysis on the orbital fat of the orbits of all participants. To this end a part of the orbital fat was manually segmented, taking care that the optic nerve and large vessels were not included. As the Dixon scans showed a relatively high water content (approximately 80%) in the orbital fat, the fat fraction was not fixed to 10% as was done for the subcutaneous fat calibration, but was a free variable of the fit.

**Result:** On average we observed a 15.9 ms lower T2_fat_ in the right orbit of the participants as compared to the subcutaneous fat of the same participant, although with a large variation between subjects (figure 2). We additionally observed that this difference was dependent on the B1 obtained from the orbital fat EPG fit (r = 0.43, p<0.0001), which is likely the result the B1-dependence of the J-coupling effects on the T2_fat_.^2–4^


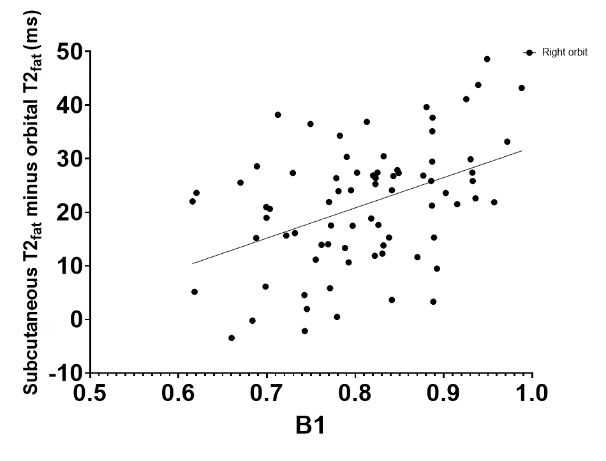


*Figure 2. Difference of the subcutaneous T2_fat_ ­of the neck and the T2_fat_ of the orbital fat in the right orbit as a function of the fitted B1 of the orbit..*

This significant difference likely explains the strong fat fraction dependence we observed in the T2_water_ fits of the EOM, as shown for lateral rectus in figure 3 on the left (r = -0.43, p < 0.0001). Given the smaller effect of B1 of the difference in T2_fat_, a less pronounced, but still significant (r = -0.25, p < 0.05), relation between T2_water_ and B1 was found for the same EOM (figure 3 right).


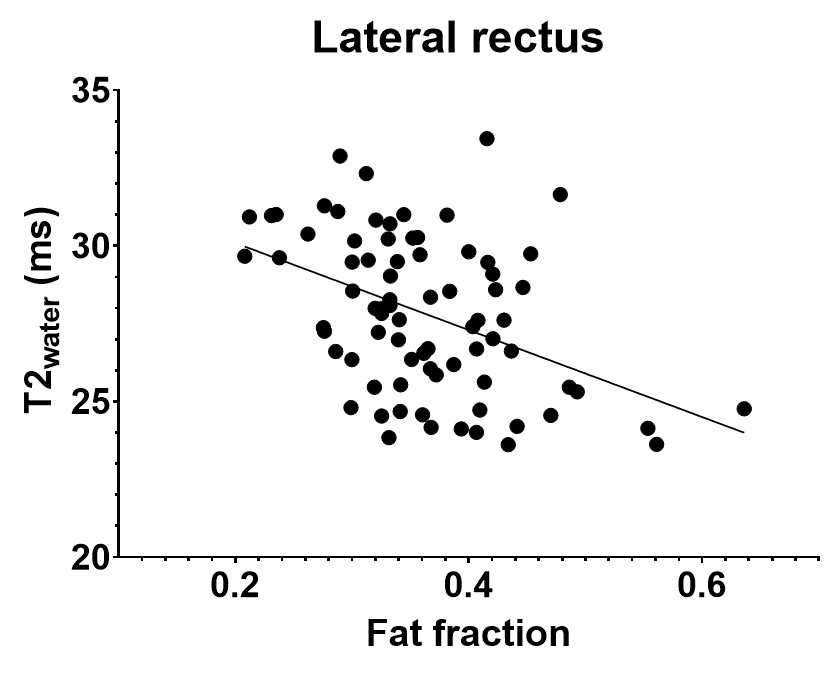

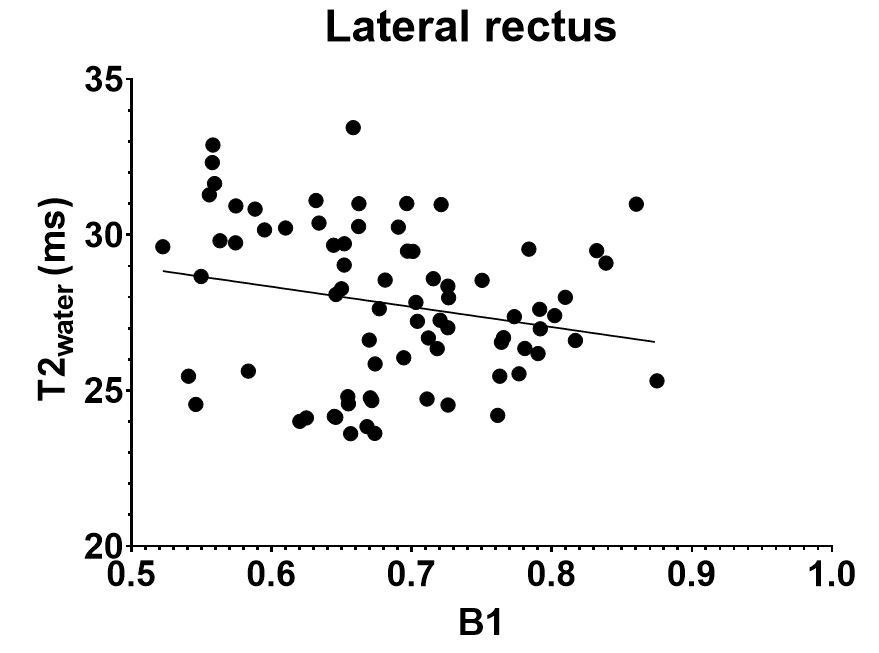


*Figure 3. Left: Correlation between the fitted fat fraction and the T2­­_water_ for the right lateral rectus. Right: Correlation between the fitted B1 and the T2_water_ for the right lateral rectus.*

**Conclusion**: A significant, 15.9 ms, difference in T2 of the subcutaneous fat and orbital fat was found, making the subcutaneous fat not representative of the fat signal which, due to the chemical shift displacement, is present in the MRI signal of the EOM.

**3) Accuracy of EPG-based B1 determination**

As in the EPG-analysis both the T2_water_ and B1 are primarily determined on the signal evolution of the first echoes, an inaccuracy in the B1 estimation will have a direct effect on the obtained T2_water_. Given the 120 mm RF-wavelength at 7 Tesla, we would not expect significant B1 variations within a cross section through an individual EOM. We therefore assessed the interquartile range (IQR) of the B1 values obtained from the approximately 80 voxels of each EOM cross section, as a proxy for the precision of the EPG-based B1 determination.


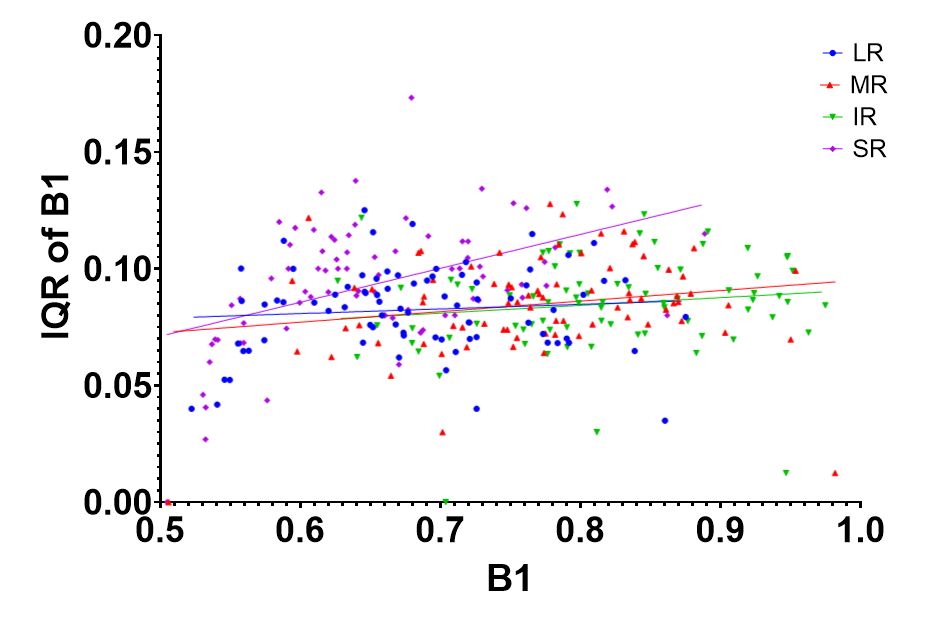

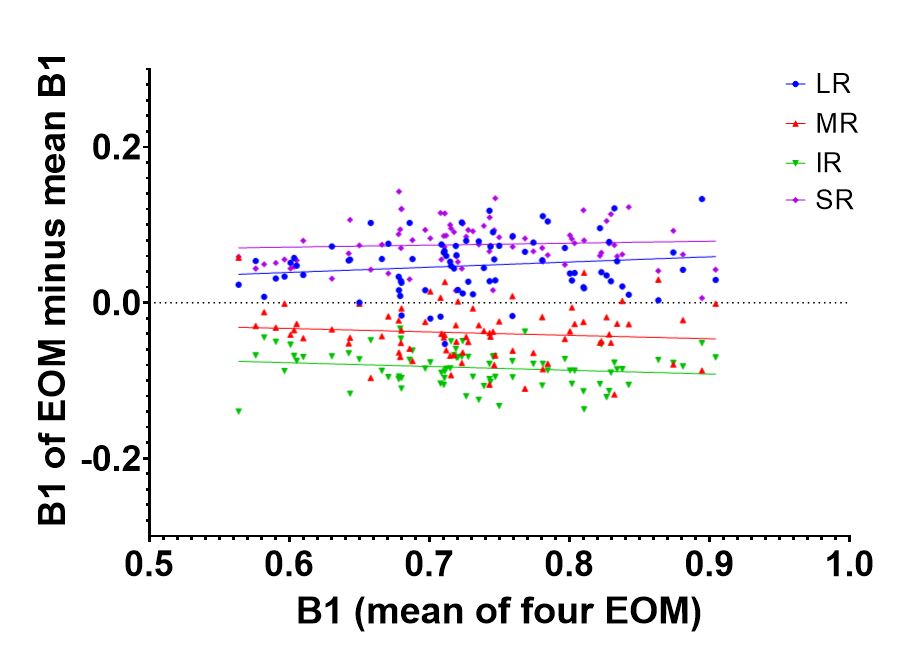


*Figure 4. Left: The interquartile range of the B1 inside the regions of interest for the EOM as a function of B1 values separated per EOM for the right orbit for all participants. Right: The mean B1 per orbit is shown on the x-axis and the mean B1 minus the B1 of an individual EOM per participant is shown on the y-axis.*

**Result**: A large IQR for the B1, on average 8%, was found, which signifies that the used echo-train has insufficient discriminative power to differentiate small changes in T2_water_ and B1 on an individual voxel level. The variation was approximately independent on EOM and B1, except at the edge of the dictionary, where, as expected, a smaller IQR was found (figure 4 left). We additionally assessed the variation in B1 between the EOM, which showed systematic differences between the EOM, with the IR achieving an on average 0.1 higher B1 than the SR (P<0.001), which was in agreement with a DREAM-B1 measurements performed in a subset of the healthy subjects.

**Conclusion:** Given the high intra-muscle variation in B1 values obtained by the EPG-analysis, a large variation in T2_water_ values is expected. A fixed B1 value should therefore be used, which should be based on the B1 of that specific muscle.

**4) Effect of noise**

To evaluate the potential effect of a (varying) signal-to-noise ratio (SNR), we assessed variation is signal obtained 27 ms after the last echo of the echo train, which was routinely acquired for all participants by disabling the RF-pulses in the last three echoes.

**Result:** In all participants the average noise floor was 0.8±0.2% of the signal magnitude from the first echo.

**Conclusion:** Measurement noise was unlikely to introduce a significant variation in the obtained B1 and T2_water­_.

**5) EOM specific EPG analysis**

Based on the results of these evaluations, we performed a modified EPG analysis of the lateral muscle of the healthy subjects, which aimed to provide a best effort, given the data available, to resolve the observed EOM and 7-Tesla specific effects:

First, for each subject an orbit specific T2_fat_ was determined, through an EPG fit of the average orbital fat signal, as obtained in section 2 above. In this fit the water content was a free fitting variable. The obtained T2_fat_ was used in all subsequent analyses of this orbit instead of the conventionally used subcutaneous fat T2. Subsequently, a “conventional” EPG fit was performed for all voxels, and based on these fits, the average B1 for each lateral rectus was calculated. Finally, a constrained EPG fit was performed in which the B1 is set to mean B1 of that muscle, as obtained in the previous step. In this fit the T2_water_ and fat fraction were the only remaining degrees of freedom.

**Result:** The fit resulted in a standard deviation of the T2_water_ of 2.5 ms, which is comparable with the standard deviation from the “conventional” EPG fit for the lateral rectus muscle (figure 5).


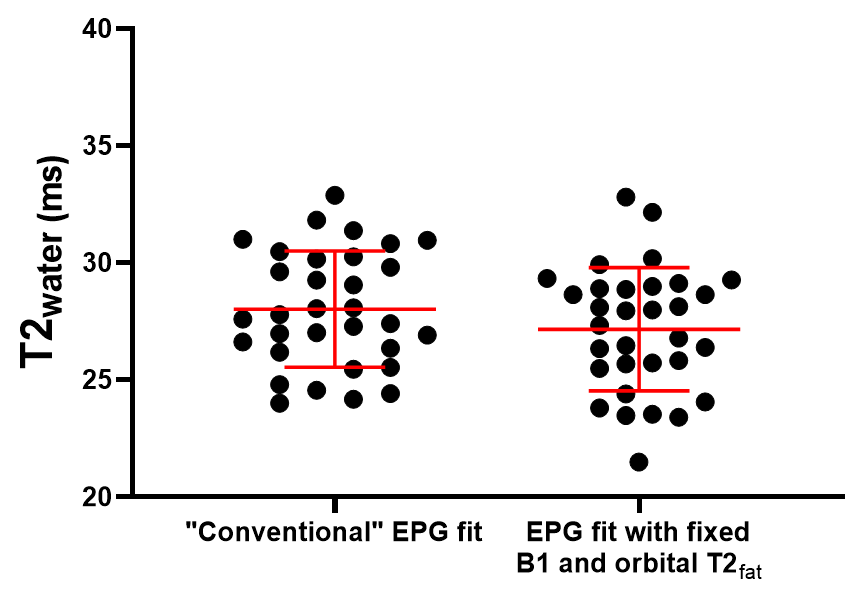


*Figure 5. The T2_water_ fitted from the “conventional” EPG fit and the proposed fit with a B1 based on the average B1 of the EOM and the T2_fat_ set to the T2_fat_ of the orbital fat for the lateral rectus muscle of the healthy controls.*

**Conclusion:** Although a high intra-muscle variation in B1 and the difference in T2 of the subcutaneous fat and orbital fat had a confounding effect on the fitted T2_water_ of the EOM, fixing the B1 and using an orbital T2_fat­_ did not lower the variation. Therefore, the origin of this high variation in T2_water_ remains unknown. It could, for example, have been a limitation of the two-component EPG model which does not incorporate a, currently unknown, EOM specific contribution to the MR-signal. The variation could also have been hardware related as, similar to a recent 3T study on intra-ocular tumours^5^, we observed a relatively low B1 in the orbit. Alternatively the T2_water_ of the EOM could indeed have had a relatively high variation between subjects, making it a less useful metric to assess subtle changes in inflammation.

**References**

1. Marty B, Baudin PY, Reyngoudt H, et al. Simultaneous muscle water T2 and fat fraction mapping using transverse relaxometry with stimulated echo compensation. *NMR Biomed*. 2016;29(4):431-443. doi:10.1002/nbm.3459

2. Hardy PA, Henkelman RM, Bishop JE, Poon ECS, Plewes DB. Why fat is bright in rare and fast spin‐echo imaging. *Journal of Magnetic Resonance Imaging*. 1992;2(5):533-540. doi:10.1002/jmri.1880020511

3. Stokes AM, Feng Y, Mitropoulos T, Warren WS. Enhanced refocusing of fat signals using optimized multipulse echo sequences. *Magn Reson Med*. 2013;69(4):1044-1055. doi:10.1002/mrm.24340

4. Froeling M, Hughes E, Schlaffke L, Kan HE, Hollingsworth KG. The relation between fat calibration in multi-echo spin-echo water T2 mapping and STEAM fat T2 relaxation measurements. In: *Proceedings of the 27th Annual Meeting of ISMRM, Montréal, Canada*. ; 2019:1273.

5. Jaarsma-Coes MG, Ferreira TA, van Houdt PJ, van der Heide UA, Luyten GPM, Beenakker JWM. Eye-specific quantitative dynamic contrast-enhanced MRI analysis for patients with intraocular masses. *MAGMA*. 2022;35(2):311-323. doi:10.1007/S10334-021-00961-W
